# Isaac Newton's description of the optic chiasm

**DOI:** 10.1055/s-0045-1814373

**Published:** 2026-01-25

**Authors:** Caio C. D. Disserol, Mario T. Sato, Yago Alfaro, Hélio A. G. Teive

**Affiliations:** 1 Universidade Federal do Paraná, Hospital de Clínicas, Departamento de Medicina Interna, Serviço de Neurologia, Curitiba PR, Brazil.; 2 Instituto de Neurologia de Curitiba, Curitiba PR, Brazil.; 3 Hospital Israelita Albert Einstein, Instituto do Cérebro, São Paulo SP, Brazil.; 4 Universidade Federal do Paraná, Hospital de Clínicas, Departamento de Medicina Interna, Serviço de Oftalmologia, Curitiba PR, Brazil.

**Keywords:** Optics and Photonics, Neurology, Vision, Ocular, Optic Nerve, Optic Chiasm

## Abstract

Sir Isaac Newton is widely regarded as one of the greatest scientific minds in history, with seminal contributions across mathematics, physics—particularly optics—and the formulation of the law of universal gravitation. Less well known, however, is his extraordinary and rarely recognized contribution to the field of neurology: the early conceptualization of fiber decussation within the optic chiasm. Through his studies on light and vision, Newton proposed that optic nerve fibers cross at the chiasm—a hypothesis that would later be anatomically confirmed and provided the basis for understanding the classic patterns of visual field deficits.

*“If I have seen more, it is by standing on the shoulders of giants”.*

**Isaac Newton** , 1676

## INTRODUCTION

Isaac Newton (1643–1727) is universally recognized as one of humanity's greatest geniuses. 1 2 His great discoveries sparked a veritable scientific revolution, spanning the fields of mathematics, physics, astronomy, and philosophy. Through the dissemination of his theories, Newton achieved international acclaim and was honored with numerous distinctions, including the title of *Knight of the British Empire* in England. 1 2 3 Very little attention, however, has been given to his contributions to neuroscience, particularly neuroanatomy, and his remarkable insight regarding the decussation of visual nerve fibers at the optic chiasm. 3 4 5 6

## ISAAC NEWTON – SHORT BIOGRAPHY

**Figure 1:**
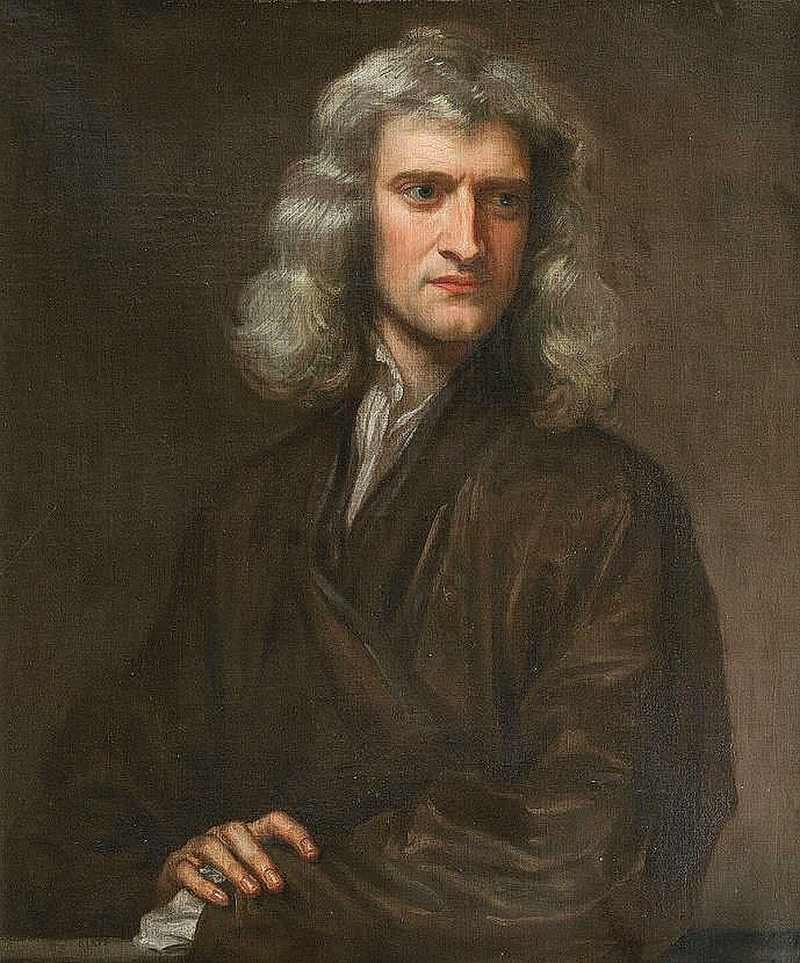
Source: Extracted from Google Images (Wikipedia), January 3rd, 2025. Sir Isaac Newton (1642–1776).

Isaac Newton ( Figure 1 ) was born on Christmas Day in 1642, in the small village of Woolsthorpe-by-Colsterworth, located in Lincolnshire, England. He was born prematurely and likely underweight for his gestational age. 1 His father, who was illiterate, died 3 months before his birth. When Newton was 3 years old, his mother remarried and entrusted his upbringing to his maternal grandmother. 1 His school career was brilliant, and he was eventually admitted to Trinity College, University of Cambrigde. There, after completing his studies, he had a blazing career, becoming Lucasian Professor of Mathematics.

Biographical accounts describe Isaac Newton as introverted, solitary, religious but not orthodox, puritanical, anxious, depressed, and celibate. He was also known to avoid social engagement. 1 His most prolific and creative period occurred between the ages of 21 and 23 years, when he carried out groundbreaking studies that led to the formulation of the basic laws of mechanics, advances in the field of optics defining the nature of light, development of calculus, as well as the creation of the law of universal gravitation, which later made him world-famous. 1 2 Newton held several bureaucratic public positions, including Member of the English Parliament, Director of the Royal Mint, and President of the Royal Society (1703). 1 2 In 1705, he was knighted by Queen Anne.

During his troubled scientific career, he engaged in notable disputes with other eminent thinkers of his time, such as Leibniz and Hooke. 1 After the age of 50 years, Isaac Newton showed symptoms of psychosis, with mercury poisoning being proposed as a potential cause. Several publications have debated whether these neuropsychiatric symptoms resulted from mercury intoxication or were manifestations of a manic-depressive illness. 1 6 7 8 9 10 11 Other hypotheses include renal lithiasis and nephrotic syndrome. 12 He died in his sleep in London in 1727, at the age of 84 years, and was buried with honors in Westminster Abbey. 1

## ISAAC NEWTON AND THE OPTIC CHIASM

Among Isaac Newton's many masterful scientific contributions is his pioneering work on light and color, particularly the refraction of light through a prism, first published in 1672. 1 2 3 The details of this seminal study were later carefully analyzed by Zemplén in a monograph published in 2005. 1 Newton's classic treatise *Opticks* , published in 1704, provides further information on this previously lesser-known field of research. 13 His specific interest in the optic chiasm deepened on March 15, 1682, following a lecture by his friend William Briggs, who taught him how to dissect the eye. 14

Nowadays, the anatomical course of the visual pathways from the retina to the occipital cortex is well established. Likewise, the formation of the optic chiasm by the partial decussation of optic nerve fibers—specifically those originating from the nasal (medial) hemiretina and running within the medial portion of the optic nerves—is now recognized as a fundamental principle in the understanding of visual field deficits. 15 Notably, this initial hypothesis that there should be a decussation of half of the optic nerve fibers at the level of the optic chiasm was not first proposed by neuro-anatomists, but rather by Isaac Newton himself, during his studies of light and optics and described in “Opticks”. 3 4 5 13

**Figure 2:**
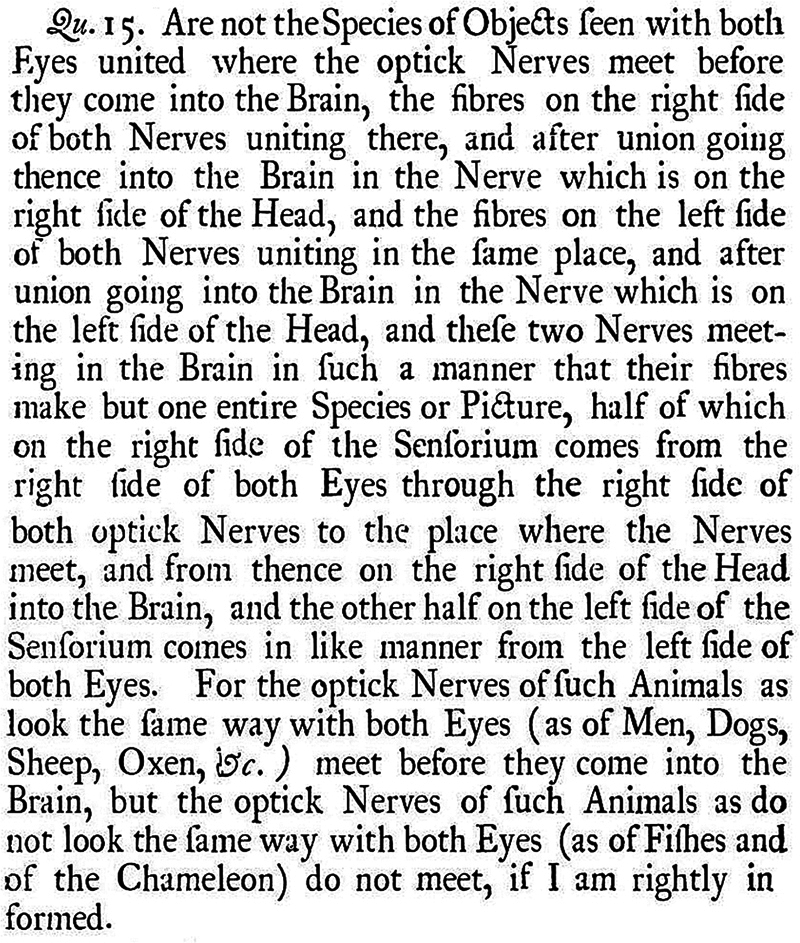
Source: Extracted from Opticks, Third Book, pages 136–137. 13 Optic chiasm description in Query 15. Caption: “Query 15. Are not the species of objects seen with both eyes united where the optic nerves meet before they enter the brain, the fibers on the right side of both nerves uniting there, and after union going thence into the brain in the nerve which is on the right side of the head, and the fibers on the left side of both nerves uniting in the same place, and after union going into the brain in the nerve which is on the left side of the head; and these two nerves meeting in the brain in such a manner that their fibers form but one entire species or picture—half of which, on the right side of the sensorium, comes from the right side of both eyes through the right side of both optic nerves to the place where the nerves meet, and from thence on the right side of the head into the brain, and the other half, on the left side of the sensorium, comes in like manner from the left side of both eyes? For the optic nerves of such animals as look the same way with both eyes (as in men, dogs, sheep, oxen, etc.) meet before they enter the brain; but the optic nerves of such animals as do not look the same way with both eyes (as in fishes and the chameleon) do not meet—if I am rightly informed.”

“Opticks” was structured into three main books followed by a series of speculative queries—philosophical and scientific hypotheses proposed by Newton to encourage further investigation. While the books detail his experiments on reflection, refraction, and diffraction, the queries, added at the end of book three, explore vision and the transmission of light through the optic nerves. 13 In Query 15, Newton suggests that the optic nerves cross at the chiasm and proposes this anatomical arrangement as the most coherent explanation for unified binocular vision ( Figure 2 ).

**Figure 3:**
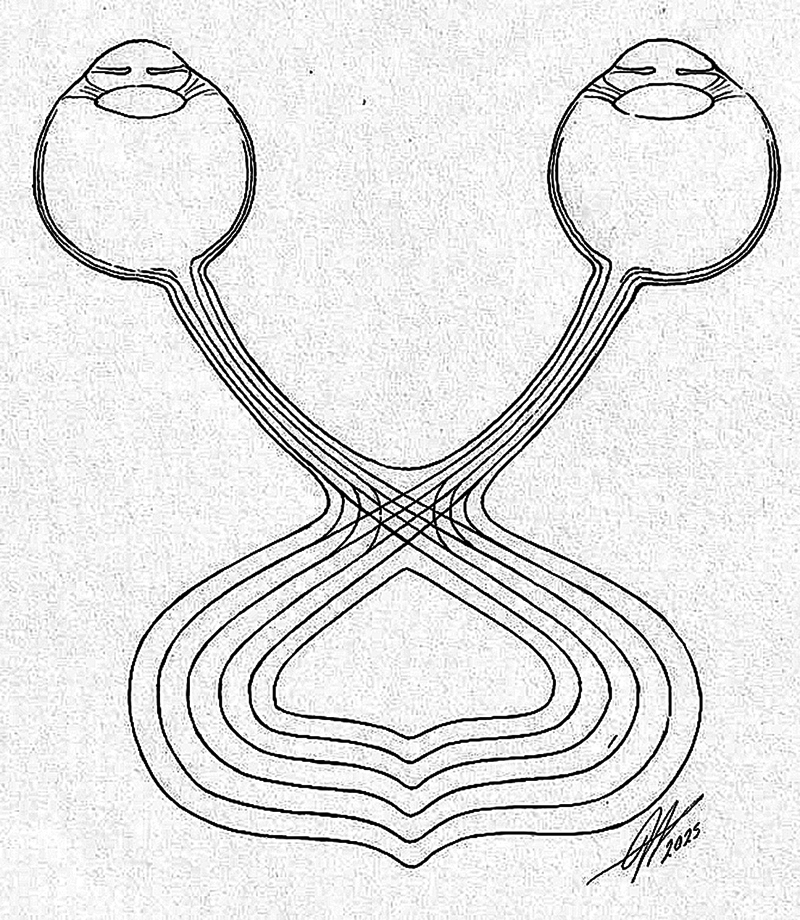
Note: Adapted from Sweeney. 3 Representation of Isaac Newton's drawing of the optic chiasm.

This description illustrates how the intuition of a genius, relying solely on technical knowledge acquired through years of research in the field of light, and without benefit of experimentation or anatomical dissection, enabled Newton to hypothesize that the optic chiasm would have crossing fibers coming from the nasal halves of each retina ( Figure 3 ). 3 5 13

Newton's hypothesis was later confirmed in 1755 by Johann Gottfried Zinn through anatomical dissections. 5 14 William Hyde Wollaston, in 1824, also confirmed these findings, based in part on his own experience with homonymous hemianopia, attributing the phenomenon to the incomplete decussation of optic nerve fibers. 14 Until then, it had been believed that the vertical meridian separating the nasal and temporal retinal fields occurred at the optic disc. 1 14

In conclusion, Sir Isaac Newton, widely regarded as one of the greatest geniuses in history, with countless scientific contributions in the fields of mathematics, defining calculus, physics, especially in optics, as well as the law of universal gravitation, made an unusual contribution to neurology. It was his initial insight that the optic chiasm had crossed fibers, offering a structural explanation for the classic patterns of visual field deficits, later confirmed by neuroanatomists.
